# The Identification and Analysis of the Self-Incompatibility Pollen Determinant Factor *SLF* in *Lycium barbarum*

**DOI:** 10.3390/plants13070959

**Published:** 2024-03-26

**Authors:** Jiali Wu, Xiongxiong Nan, Xin Zhang, Wendi Xu, Haijun Ma, Zijun Yang, Cuiping Wang

**Affiliations:** 1 School of Biological Science and Engineering, North Minzu University, Yinchuan 750021, China; wujiali15025787340@163.com (J.W.);; 2 State Key Laboratory of Efficient Production of Forest Resources, Yinchuan 750004, China; 3 Innovation Team for Genetic Improvement of Economic Forests, North Minzu University, Yinchuan 750021, China; 4 Ningxia Grape and Wine Innovation Center, North Minzu University, Yinchuan 750021, China

**Keywords:** *Lycium barbarum*, self-incompatibility, pollen determinant factor, *F-box* gene family, S-locus *F-box* gene

## Abstract

Self-incompatibility is a widespread genetic mechanism found in flowering plants. It plays a crucial role in preventing inbreeding and promoting outcrossing. The genes that control self-incompatibility in plants are typically determined by the S-locus female determinant factor and the S-locus male determinant factor. In the Solanaceae family, the male determinant factor is often the *SLF* gene. In this research, we cloned and analyzed 13 *S_2_-LbSLF* genes from the *L. barbarum* genome, which are located on chromosome 2 and close to the physical location of the S-locus female determinant factor S-RNase, covering a region of approximately 90.4 Mb. The amino acid sequence identity of the 13 *S_2_-LbSLFs* is 58.46%, and they all possess relatively conserved motifs and typical *F-box* domains, without introns. A co-linearity analysis revealed that there are no tandemly repeated genes in the *S_2_-LbSLF* genes, and that there are two pairs of co-linear genes between *S_2_-LbSLF* and the tomato, which also belongs to the Solanaceae family. A phylogenetic analysis indicates that the *S_2_-LbSLF* members can be divided into six groups, and it was found that the 13 *S_2_-LbSLFs* are clustered with the *SLF* genes of tobacco and *Petunia inflata* to varying degrees, potentially serving as pollen determinant factors regulating self-incompatibility in *L. barbarum*. The results for the gene expression patterns suggest that *S_2_-LbSLF* is only expressed in pollen tissue. The results of the yeast two-hybrid assay showed that the C-terminal region of *S_2_-LbSLFs* lacking the *F-box* domain can interact with S-RNase. This study provides theoretical data for further investigation into the functions of *S_2_-LbSLF* members, particularly for the identification of pollen determinant factors regulating self-incompatibility in *L. barbarum*.

## 1. Introduction

*Lycium barbarum*, a species of the genus *Lycium* within the Solanaceae family, is a perennial shrub primarily distributed in Ningxia, Xinjiang, Inner Mongolia, but is also found in other regions. The *L. barbarum* fruit is valued for its medicinal and culinary benefits, including its ability to lower blood pressure, nourish the kidneys and lungs, protect the liver, and replenish blood, as well as its whitening properties [1,2]. In recent years, *L. barbarum* has become increasingly popular on the market, leading to a continuous expansion of planting areas and the development of many high-yielding and high-quality varieties. However, most of these varieties still need to be planted with others to facilitate cross-pollination for normal fruit setting, and self-incompatibility is the underlying cause of issues like inconsistent fruit sizes, severe flower and fruit drop, and even complete crop failure when single varieties are grown over large areas [3]. Studies on the breeding systems and self-incompatibility of various *L. barbarum* varieties have revealed that, aside from ‘Ningqi 1’ and ‘Ningqi 7’, which have higher self-compatibility, most other varieties are predominantly self-incompatible or have very low compatibility. As a result, self-compatibility has emerged as a crucial factor for *L. barbarum* breeders to consider when selecting new varieties.

Self-incompatibility (SI) is a genetic mechanism that is widespread in many flowering plants [4,5] that helps to mitigate the negative effects of inbreeding by preventing self-fertilization and promoting cross-pollination [6]. This mechanism is usually governed by a single S locus, which comprises at least two genes that are closely associated and exhibit tissue specificity, encoding the male (pollen) and female (stigma) S-determinants [7,8]. Although research has shown that plant self-incompatibility is regulated by the *S-RNase* gene (responsible for stigma determinants) and the *SLF* (*S*-*locus F-box*) gene, which encodes F-box proteins, in families such as Solanaceae, Plantaginaceae, and Rosaceae [9,10,11], studies have revealed that the functions of *SLF*/*SFB* genes vary among these families. In Rosaceae, *SLF* gene mutations or losses in the Prunus genus can lead to the self-S-RNase accepting pollen, whereas in Solanaceae and Plantaginaceae, *SLF* genes act as degradative factors to break down non-self S-RNase [5,12]. For instance, Sijacic et al. identified and characterized *S-locus F-box* genes in *Petunia hybrida* and found that pollen carrying different S alleles can lose their self-incompatibility, becoming self-compatible [13]. Qiao et al. also discovered, in snapdragons, that when *AhSLF-S_2_* was overexpressed in homozygous S_3_S_3_ plants, only pollen carrying both *AhSLF-S_2_* and S_3_ could grow normally [14]. In Solanaceae *L. barbarum* plants, self-incompatibility is mediated by a class of ribonucleases (S-RNases), where the recognition of self/non-self between pollen and stigma is determined by highly polymorphic S loci. When both express the same S specificity, the pollen is rejected by the stigma. The S-RNase is considered cytotoxic, and when it enters the self-pollen tubes, it can degrade self-pollen-tube RNA [15], inhibiting pollen tube growth [16,17,18] and leading to self-incompatibility. The *SLF*/*SFB* genes closely associated and interacting with S-RNase can ubiquitinize non-self S-RNase through the formation of the *SCF* complex, and the ubiquitinated S-RNase is eventually degraded by the 26S proteasome [19,20,21], thereby eliminating the cytotoxic effects of S-RNase and allowing the pollen tube to grow normally in the stigma and complete fertilization.

To date, numerous stigma-specific genes, known as S-RNases, have been isolated from various self-incompatible plants, including Solanaceae, Plantaginaceae, and Rosaceae [22], and their mechanisms of action have been investigated in considerable depth. However, research into the key pollen-control genes has lagged behind. Lai et al. were the first to discover the *S-RNase* gene by sequencing DNA fragments containing the *S-RNase* gene region in the genomes of snapdragons and identified a closely linked *F-box* gene, *AhSLF-S_2_*, which is specifically expressed in pollen [23]. Subsequently, similar pollen-specific *S*-*locus F-box* genes were detected in Rosaceae plants [24,25,26] and Solanaceae plants [13,14,27]. In 2004, *PiSLF-S_2_* was identified in *P. inflata* and was shown to play a role in controlling pollen self-incompatibility [14,28,29]. In 2007, Wheeler et al. discovered seven *S-locus F-box* genes in tobacco that were specifically expressed only in pollen [30]. These genes are collectively referred to as *SLF* genes in Solanaceae and Plantaginaceae or *SFB* genes in Rosaceae [31]. The N-terminus of *SLF* genes features an F-box domain, while the C-terminus possesses two highly variable regions (Hva and Hvb) that lack strong hydrophobicity and are primarily responsible for recognizing self and non-self *S-RNase* genes [32].

Pollen specificity is not governed by a single *SLF* gene, but rather by multiple *SLF* genes that collaborate (the precise number remains unknown) [33,34]. Kubo et al. first introduced a model of multiple *SLF* genes working together to recognize non-self in *P. inflata*, and they demonstrated through immunocytochemical assays that each SLF protein can interact with various S-RNase proteins across different S-haplotypes [16]. Subsequently, in 2015, a mathematical model validated that between 16 and 20 *SLF* genes on the S-haplotype of *P. inflata* are responsible for recognizing the majority of non-self S-RNase proteins [35]. To date, the same 17 polymorphic *SLF* genes have been identified in both the S2 and S3 haplotypes of *P. inflata* [13,36], suggesting that multiple *SLF* genes serve as pollen S-determinants on the S-haplotypes of *P. hybrida*. In each species, a comprehensive set of SLF proteins is necessary to counteract the toxicity of non-self S-RNase, enabling compatible pollination. However, there have been no studies on the pollen-specific expression of *F-box* genes in *L. barbarum*. To comprehend the regulatory factors of self-incompatible pollen in *L. barbarum*, it is imperative to identify all the *SLF* genes that are involved in pollen specificity within *L. barbarum*.

In our prior studies, we developed a hybrid F1 population by mating plants with contrasting phenotypes through the crossing of plants exhibiting extreme phenotypes. We employed a bulked segregant analysis (BSA) to locate the S locus on chromosome 2 in *L. barbarum*. Subsequent analyses, involving expression profiling and population genotyping, indicated that the S-RNase is a potential determinant of self-incompatibility in the female flowers of *L. barbarum* [37]. Recent studies on the *F-box* gene family in *L. barbarum* have uncovered that 13 *F-box* genes located on chromosome 2 are exclusively expressed in the stamen and are physically located close to the *S-RNase* gene (*Lba02g01102*), indicating a strong association with S-RNase. These genes are believed to function as stamen S-determinants, potentially contributing to the collective decision-making process of self-incompatibility recognition in *L. barbarum*. To explore the possibility that these stamen S-determinants are involved in self-incompatibility, in the current study, we cloned these 13 *F-box* genes and conducted a thorough analysis of their gene structures, conserved protein domains, motifs, and regulatory elements, such as promoters and trans-acting factors, using bioinformatics tools. An evolutionary analysis was also conducted to categorize these genes and identify any co-linear genes. The objective was to elucidate the pollen-specific expression patterns of *F-box* genes and their interactions with the female determinant S-RNase. This research not only yields theoretical insights into the function of the self-incompatibility stamen determinant *S_2_-LbSLF* genes but also provides molecular markers that could be useful in the breeding of self-compatible *L. barbarum* varieties.

## 2. Results

### 2.1. Identification and Cloning of the F-Box Gene Family in L. barbarum

**Figure 1:**
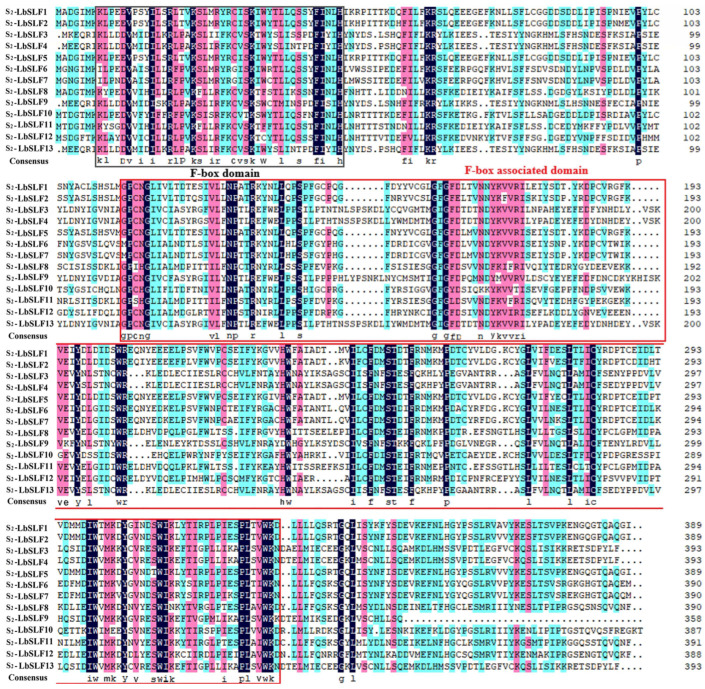
The amino acid sequence alignment of the *S_2_-LbSLFs*. The black-boxed regions represent the F-box structure domain, and the red-boxed regions represent the *F-box*-associated domain (F-box-associated domain). The conservation strength of amino acids from strong to weak is highlighted with dark blue, purple, and light blue, respectively.

In the *L. barbarum* genome, a total of 283 genes belonging to the *F-box* gene family were identified, distributed unevenly across 12 chromosomes, with the highest number found on chromosome 2, which harbors 40 genes. Since the stamen determination factor and the pistil determination factor are located at the S locus, we successfully cloned an *S-RNase* gene encoding the pistil determination factor from *L. barbarum*, which is located on chromosome 2. It is hypothesized that the male determination factor responsible for self-incompatibility in *L. barbarum* is also located on chromosome 2. Additionally, 13 *F-box* genes on chromosome 2 were found to be specifically expressed in the stamens, suggesting that these genes may collectively regulate the self-incompatibility of *L. barbarum* in conjunction with the S-RNase. Consequently, these 13 *F-box* genes on chromosome 2 were cloned to obtain the sequences of the *S_2_-LbSLFs*, which were named *S_2_-LbSLF1–S_2_-LbSLF13*. The coding sequences of the 13 *SLF* genes in *L. barbarum* typically range in length around 1150 base pairs, and the SLF proteins all possess F-box- and F-box-associated domains (F-box-associated) (Figure 1). The 13 *SLF* genes are highly similar to one another, exhibiting 58.46% amino acid sequence identity.

### 2.2. Physicochemical Properties, Subcellular Localization Prediction, and Functional Prediction of S_2_-LbSLFs

Utilizing the ExPASy-ProtParam tool to determine protein characteristics such as length, molecular weight, isoelectric point (pI), instability index, lipophilic index, and hydrophilic index (Table 1), the findings indicate that the *S_2_-LbSLFs* vary in amino acid length from 358 (*S_2_-LbSLF9*) to 393 (*S_2_-LbSLF3*, *S_2_-LbSLF4*) amino acids. The predicted molecular weights range from 42,018.48 (*S_2_-LbSLF9*) to 45,805.57 (*S_2_-LbSLF4*) Da. The predicted pI values for the *S_2_-LbSLFs* range from 4.81 (*S_2_-LbSLF1*) to 7.07 (*S_2_-LbSLF9*), with *S_2_-LbSLF4* and *S_2_-LbSLF10* predicted to have pI values above 7, suggesting that they may be basic proteins. The instability indices predict that all the proteins except *S_2_-LbSLF6* and *S_2_-LbSLF7* have indices above 40, indicating potential instability. The lipophilic indices are predicted to range from 86.34 (*S_2_-LbSLF10*) to 99.41 (*S_2_-LbSLF8*), while the hydrophilic indices for the *S_2_-LbSLFs* are expected to range from −0.007 (*S_2_-LbSLF8*) to −0.248 (*S_2_-LbSLF9*), with all falling below 0, suggesting hydrophilic properties that all *S_2_-LbSLFs* have. The subcellular localization predictions using the Cell-PLoc 2.0 online tool suggest that all 13 S_2_-LbSLF proteins are located in the nucleus.

The secondary structure analysis of the 13 S_2_-LbSLF proteins reveals a composition of four structural types: alpha helix, extended strand, beta turn, and random coil. The alpha helix and random coil structures dominate, accounting for over 60%, while the extended strand and beta turn structures account for approximately 30%. The random coil structure is the most abundant, comprising around 50% of the structure, followed by the extended strand (Table 2).

### 2.3. Conservation Motifs, Structural Domains, and Gene Structure of S_2_-LbSLF Gene Members

**Figure 2:**
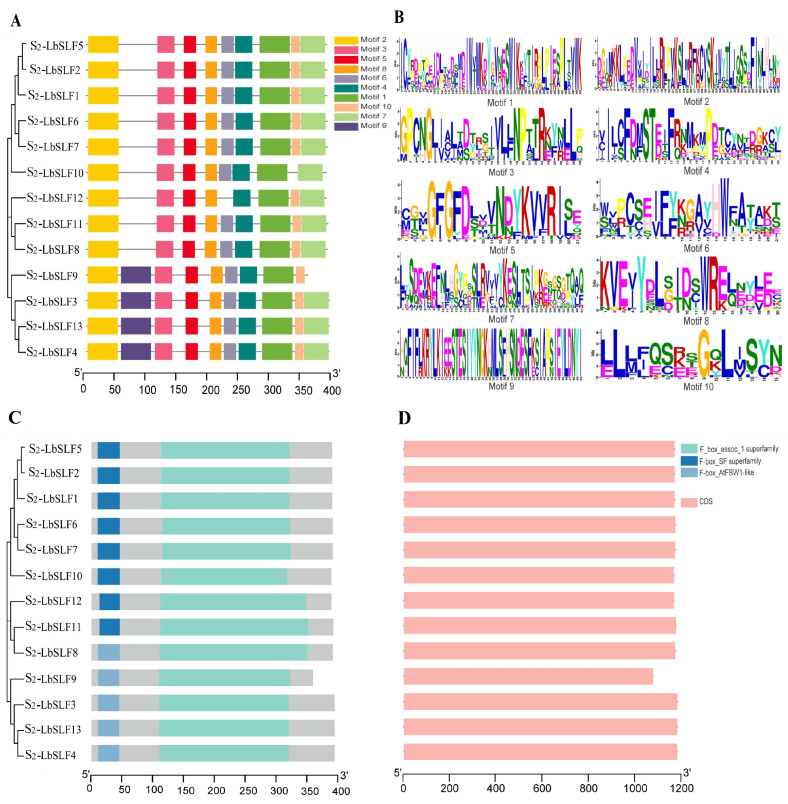
Analysis of conserved motifs, domains, and gene structure of *S_2_-LbSLF* gene members. (**A**) Analysis of conserved motifs in S_2_-LbSLFs proteins, where 10 conserved motifs were identified. (**B**) Sequence logos of the 10 conserved motifs. The motif logo is composed of a stack of letters at each position, with the relative size of the letters indicating their frequency in the sequence. (**C**) Conserved domains in S_2_-LbSLFs proteins, with non-domain regions represented in gray. (**D**) *S_2_-LbSLFs* gene structure: exons–introns.

The analysis using the MEME online tool revealed that the *S_2_-LbSLF* genes in *L. barbarum* have 10 relatively conserved motifs, with lengths varying from 15 to 50 amino acids (Figure 2B). All 13 members of the S_2_-LbSLFs proteins possess Motif1, Motif2, Motif3, Motif4, Motif5, and Motif8, indicating that these six motifs are highly conserved. The *S_2_-LbSLF3*, *S_2_-LbSLF4*, *S_2_-LbSLF9*, and *S_2_-LbSLF13* have an additional Motif9 compared to the other *S_2_-LbSLFs*, while *S_2_-LbSLF9*, *S_2_-LbSLF10*, and *S_2_-LbSLF12* lack Motif7, Motif10, and Motif6, respectively (Figure 2A). The conserved regions of the S_2_-LbSLF family members show that all 13 S_2_-LbSLF proteins have the typical F-box protein structure, with an N-terminal F-box domain involved in interaction with Skp1 and a C-terminal region involved in interaction with substrate proteins, belonging to the F_box_assoc_1 superfamily (Figure 2C). To understand the structure and genetic diversity of the *S_2_-LbSLFs* genes, an exon–intron structure analysis and visualization were performed using the TBtools (v2.042) software, and it was found that none of the *S_2_-LbSLFs* contain introns (Figure 2D).

### 2.4. Analysis of Promoter Cis-Acting Elements

**Figure 3:**
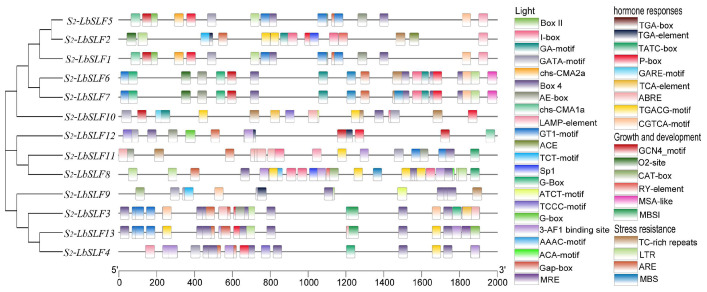
Analysis of cis-acting elements in the promoter region of *S_2_-LbSLF* family members. The colored boxes indicate the types of cis-elements. Different colors represent different numbers of cis-elements.

Promoter cis-acting elements are critical binding sites for transcription initiation and play a pivotal role in regulating gene expression. In *L. barbarum*, the upstream 2000-base-pair promoter regions of the 13 *S_2_-LbSLF* family members are extensively annotated, and predictions have uncovered 41 cis-acting elements with potential capabilities for environmental and hormonal responses (Figure 3). These elements encompass photoresponsive elements, various factors related to growth and development, hormone response, and stress resistance (Supplementary Table S1). Many elements associated with hormone-signaling pathways were identified, including drought-response elements and methyl jasmonate (MeJA)-, abscisic-acid-, salicylic-acid-, auxin-, and gibberellin-response elements. Elements related to stress resistance include defense and stress response, cell cycle regulation, anaerobic response, and low-temperature-response elements. The GCN4_motif, O2-site, CAT box, RY-element, MSA-like, and MBSI elements are linked to growth and development. There is considerable diversity in the types and distribution of cis-acting elements within *S_2_-LbSLF* promoters. Specifically, 12 *S_2_-LbSLFs* contain light-related elements (G-Box), and the predictions also revealed MeJA-response elements (TGACG-motif and CGTCA-motif) in all the promoters except for *S_2_-LbSLF12*. Furthermore, MYB-binding sites (MBS) were found in the promoters of *S_2_-LbSLF6*, *S_2_-LbSLF7*, and *S_2_-LbSLF8*, which are associated with drought response. Salicylic-acid-related elements (TCA-element) are exclusively present in the promoters of *S_2_-LbSLF3*, *S_2_-LbSLF10*, and *S_2_-LbSLF13*. Cell-cycle-response elements (MSA-like) were only found in the promoters of *S_2_-LbSLF6* and *S_2_-LbSLF7*.

### 2.5. Phylogenetic Analysis and Classification of the L. barbarum S_2_-LbSLF Gene Family

**Figure 4:**
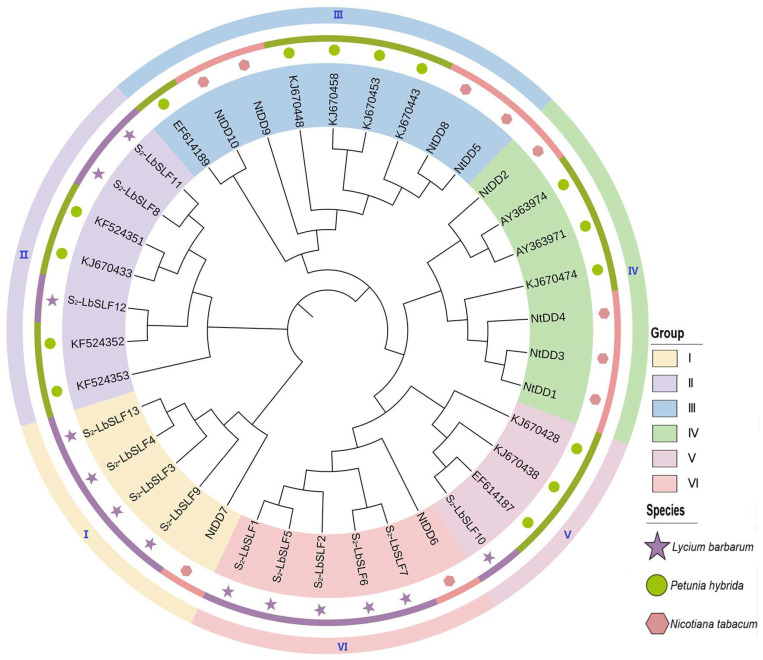
Phylogenetic tree of F-box protein family of *L. barbarum*, *N. tabacum*, and *P. inflata*. A maximum likelihood (ML) phylogenetic tree was constructed with neighbor joining (NJ) based on bootstrap sampling in MEGA11. Each color region represents a group. The same species are displayed in stripes of the same color. Stars indicate *F-box* identified in *L. barbarum*.

To delve into the similarity and evolutionary connections between the *S_2_-LbSLFs* in *L. barbarum*, a phylogenetic evolutionary tree was constructed using amino acid sequences from 13 individuals from *L. barbarum*, 15 from *P. inflata*, and 10 from *Nicotiana tabacum*. The branch clustering results led to the division of the S_2_-LbSLFs family into six groups (I–VI) (Figure 4), comprising five, seven, nine, seven, four, and six members, respectively. Group I encompasses four *L. barbarum* SLFs (*S_2_-LbSLF3*, *S_2_-LbSLF4*, *S_2_-LbSLF9*, and *S_2_-LbSLF13*) along with one *N. tabacum* SLF (*NtDD7*), suggesting a closer evolutionary relationship between these four SLFs and *NtDD7*; Group II includes three *L. barbarum* SLFs (*S_2_-LbSLF8*, *S_2_-LbSLF11*, and *S_2_-LbSLF12*) and four *P. inflata* SLF family members clustered together; Group V contains one *L. barbarum* SLF (*S_2_-LbSLF10*) and three *P. inflata* SLFs, with *S_2_-LbSLF10* clustering with *EF614187*; and Group VI consists of five *L. barbarum* SLF members (*S_2_-LbSLF1*, *S_2_-LbSLF2*, *S_2_-LbSLF5*, *S_2_-LbSLF6*, and *S_2_-LbSLF7*), along with one *N. tabacum* SLF (*NtDD6*). The clustering analysis revealed that, with the exception of Groups III and IV, which lack *L. barbarum* SLF members, the remaining four groups all feature members from the families *L. barbarum*, *N. tabacum*, or *P. inflata*. Wheeler et al. established through research on *N. tabacum* that *NtDD6* and *NtDD7* are specifically expressed in pollen, with *NtDD7* considered a potential SLF homolog [30]. This evolutionary tree suggests that certain candidate *F-box* genes in *L. barbarum* (*S_2_-LbSLF1*, *S_2_-LbSLF2*, *S_2_-LbSLF3*, *S_2_-LbSLF4*, *S_2_-LbSLF5*, *S_2_-LbSLF6*, *S_2_-LbSLF7*, *S_2_-LbSLF9*, and *S_2_-LbSLF13*) may act as pollen S-determinants and participate in self-incompatibility responses. Previous studies indicated that seven species of *P. inflata* SLF family members (*AAS79485*, *KJ670474*, *EF614187*, *KF524351*, *KF524352*, *KF524353*, and *EF614188*), which have been genetically modified and functionally validated for encoding pollen-specific determinants [13,16,36], clustered with *KF524351*, *KF524352*, *KF524353*, and *EF614187* in Groups II and V, further indicating that *S_2_-LbSLF8*, *S_2_-LbSLF10*, *S_2_-LbSLF11*, and *S_2_-LbSLF12* are promising candidates for pollen determinants.

### 2.6. Density and Collinearity Analysis of S_2_-LbSLF Genes

**Figure 5:**
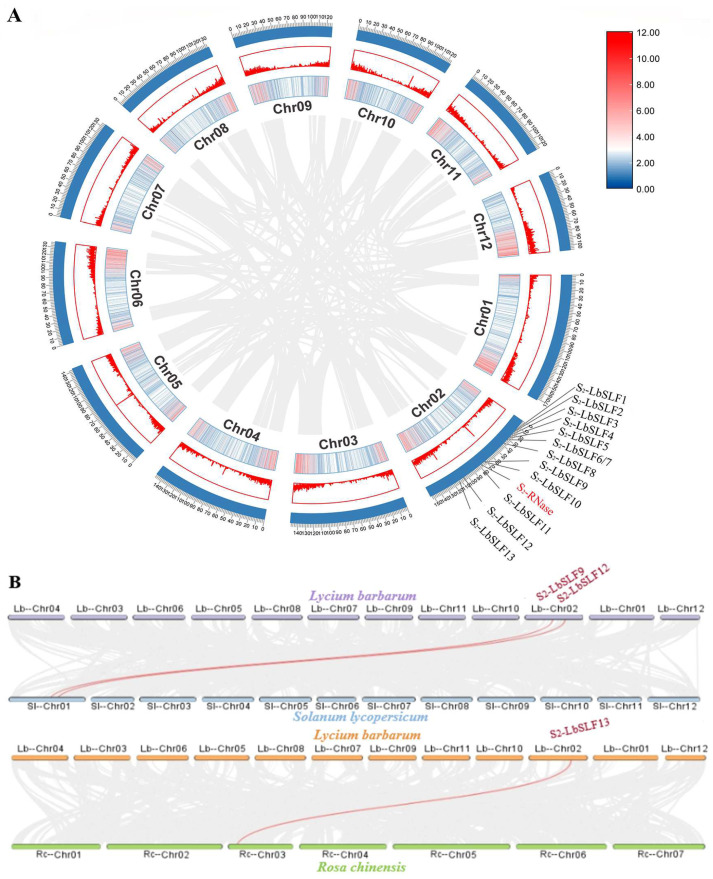
Localization of *S_2_-LbSLFs* in *L. barbarum* and collinearity with *S. lycopersicum* and *R. chinensis* chromosomes. (**A**) Thirteen *S_2_-LbSLFs* are located on chromosome 2. The scale on the outer circle is measured in megabytes. The inner and middle circles show gene density by color or column height. These connections indicate gene tandem. (**B**) Collinearity of the chromosomes of *L. barbarum* with those of *S. lycopersicum* and *R. chinensis*. These associations indicate a relationship with collinear genes. The labeled *S_2_-LbSLFs* are collinear with their SLF connected with a red line. The remaining unlabeled *S_2_-LbSLFs* were identified as non-collinear genes of *SlSLFs* or *RcSLFs*.

A collinearity analysis was performed on the *L. barbarum* genome, resulting in the placement of the 13 *S_2_-LbSLFs* and the *S-RNase* on the second chromosome of *L. barbarum*. To ascertain whether the neighboring genes were tandem duplicates, a gene duplication analysis was conducted on the *L. barbarum* genome using Tbtools (Figure 5A). The findings revealed that the identified *S_2_-LbSLFs* did not exhibit evidence of tandem duplication. There were two pairs of colinear genes observed between *L. barbarum* and *Solanum lycopersicum*, as well as one pair between *L. barbarum* and *Rosa chinensis*, with *S_2_-LbSLF9*, *S_2_-LbSLF12*, and *S_2_-LbSLF13* being the specific genes in *L. barbarum* (Figure 5B). Upon examination of the collinearity results, it was observed that there was a higher degree of collinearity between *L. barbarum*, a member of the Solanaceae family, and *S. lycopersicum.*

### 2.7. Analysis of Pollen-Specific Expression Patterns of S_2_-LbSLFs Genes

**Figure 6:**
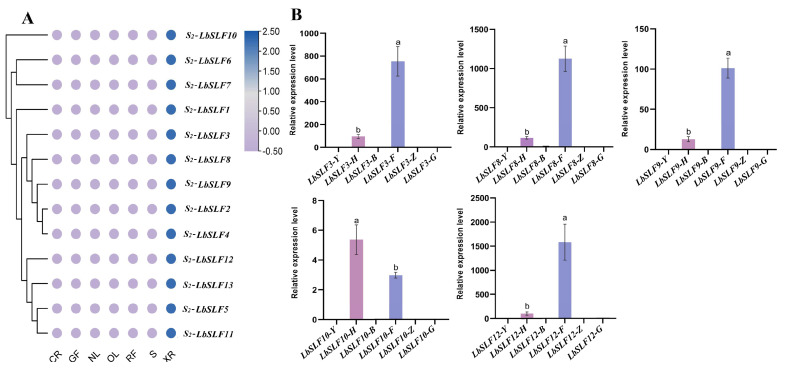
Analysis of gene expression patterns of *S_2_-LbSLFs*. (**A**) Heatmap showing the relative expression levels of genes in the *L. barbarum* transcriptome. CR: carpel; XR: anther; S: stem tip; OL: old leaf; NL: new leaf; GF: green fruit; RF: mature fruit. (**B**) Relative expression levels of *S_2_-LbSLF* genes in different tissues of *L. barbarum*. Y: leaf; H: entire flower; B: petal; F: pollen; Z: stigma; G: flower stalk. Each column represents the mean ± SD from three biological replicates, with error bars denoting the standard deviation. Different lowercase letters indicate significant differences in the relative expression levels (*p* < 0.05).

Gene expression patterns can offer crucial insights into gene function. To explore the tissue-specific expression patterns of *S_2_-LbSLF* genes, we conducted a dedicated analysis of the *F-box* genes located on the second chromosome and produced a heatmap representing their expression profiles. Our analysis revealed that the transcriptomic data from various tissues pointed to the specific expression of 13 *F-box* genes in pollen (Figure 6A). To corroborate the integrity of the transcriptomic data, we selected five genes for Qrt-PCR validation. The results aligned with our predictions, demonstrating that the five *S_2_-LbSLFs* genes were indeed specifically expressed in pollen (Figure 6B), which confirms the anther-specific expression of *S_2_-LbSLFs* genes. These results suggest that *F-box* genes with elevated expression in particular tissues are most probably engaged in tissue-specific biological processes, and further suggest that these 13 *S_2_-LbSLFs* genes may function as pollen determinants involved in the modulation of *L. barbarum*’s self-incompatibility mechanisms.

### 2.8. Interaction Analysis of SLF and S-RNase

**Figure 7:**
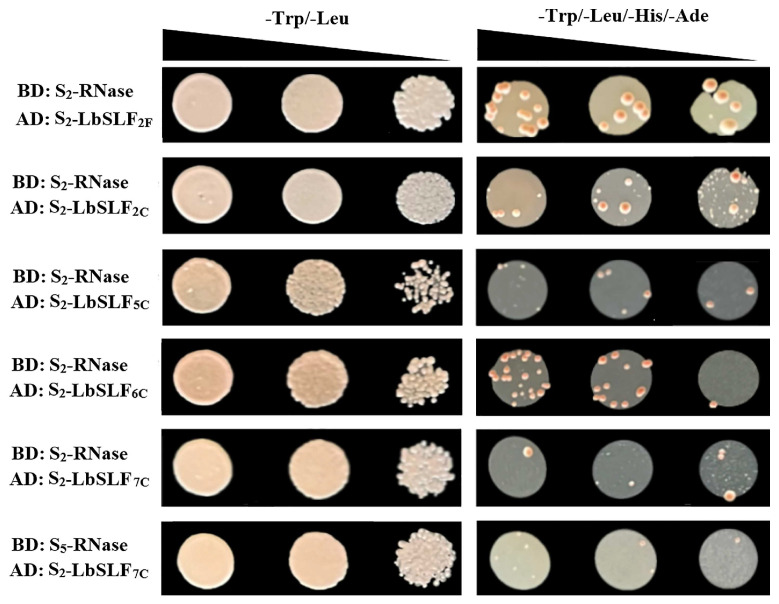
The interaction between *S_2_-RNase* and *S_5_-RNase* with *S_2_-LbSLFs* was investigated in yeast strains. AD: S_2_-LbSLFF and S_2_-LbSLFC denote the full-length and C-terminal regions of *S_2_-LbSLF*, respectively, with the numbers indicating the specific genes. BD: S-RNase signifies *S_2_-RNase* and *S_5_-RNase*. The growth of yeast cells expressing various fusion combinations of BD and AD was assessed on -Trp/-Leu- and -Trp/-Leu/-His/-Ade-minimal media lacking the specified amino acids.

The *S-RNase* and *SLF/SFB* genes have been shown to control the specificity of female organ and pollen self-incompatibility, respectively, with their protein products interacting to determine self-/non-self-specific recognition between female organs and pollen. Furthermore, SLFs can form SCF (SKP1/Cullin1/*F-box*) complexes, functioning as specific E3 ubiquitin ligases that interact with S-RNase. To elucidate the interaction between SLF and S-RNase, a yeast two-hybrid pair-wise interaction assay was employed to study the interaction between S-RNase and SLF. The *S_2_-LbSLF1–S_2_-LbSLF13* genes were segmented into full-length, N-terminal (including *F-box* domain), and C-terminal (*F-box* associated domain) regions, which were individually tested for interaction with *S_2_-RNase* and *S_5_-RNase* using yeast two-hybrid (Y2H) experiments. The *S_2_-RNase* and *S_5_*-*RNase* were amplified from the entire flower number 6 (S_2_S_5_). The interactions were observed through growth on -Leu/-Trp-defective medium and -Ade/-His/-Leu/-Trp-defective media (Supplementary Figures S1–S6). The results revealed that the full-length *S_2_-LbSLF1–S_2_-LbSLF13* did not interact with *S_5_*-*RNase*, and the C-terminal region of *S_2_-LbSLF7* could interact with *S_5_-RNase*. The full-length and C-terminal regions of *S_2_-LbSLF2*, as well as the C-terminal region of *S_2_-LbSLF5*, *S_2_-LbSLF6*, and *S_2_-LbSLF7*, could interact with *S_2_*-*RNase* (Figure 7).

## 3. Discussion

Solanaceous plants typically exhibit gametophytic self-incompatibility, which is mediated by S-RNase. The female S-determinant is encoded by a class of glycoproteins with ribonuclease activity, known as S-ribonuclease or S-RNase [38], while the pollen S-determinant is encoded by genes containing an F-box structure, referred to as S-locus F-box (SLF) [25]. The F-box proteins generally serve as components of the SCF (SKP1/Cullin1/F-box) ubiquitin ligase complex, which is responsible for mediating the ubiquitination and degradation pathway [39]. The N-terminal region of F-box proteins, typically composed of 40–50 amino acids, acts as the binding site for Skp1- or Skp1-like proteins within the SCF complex. The C-terminal domain of these proteins has the capacity to specifically recognize substrates.

### 3.1. Cloning of Full-Length Sequences for 13 SLF Genes Located on Chromosome 2 in L. barbarum

In Solanaceous plants, the interaction between pollen and pistil is a complex non-self-recognition process, as evidenced by the functional gain-of-function and loss-of-function experiments conducted by Lee et al. [40]. The female-specific determinant is encoded by a single polymorphic *S-RNase* gene, whereas multiple polymorphic *SLF* genes collectively encode the pollen-specific determinant [16,41]. For instance, in *P. inflata*, approximately 3.1 Mb of the S locus have been cloned to uncover 17 *SLF* genes and *SLFLike1*. In citrus, each S haplotype on chromosome 1 harbors one S-RNase and nine SLFs, spanning a region of 198–370 kb [10]. In roses, 30 SLFs are specifically located on chromosome 3, occupying a region of 1.5 to 43.7 Mb [42]. A study on potato chromosome 1 found that the S locus contains 13 SLFs, covering an area of 14.6 Mb [43]. In snapdragons, chromosome 8 contains 32 SLF proteins, spanning 1.2 Mb [44]. However, to date, no published studies have reported *SLF* genes located on the chromosomes of *L. barbarum*. In previous work, we cloned the *S_2_-RNase* gene, which is an estigmatic determinant regulating self-incompatibility in *L. barbarum*. Therefore, in this study, we aimed to clone and analyze the SLF pollen determinant located near the S-RNase on chromosome 2 of *L. barbarum*. The cloned region spans approximately 90.4 Mb of the S locus on this chromosome. Compared to other species, Solanaceous plants have a larger area covered by the S locus, which may be due to their rich, highly repetitive sequences [45]. Through the sequence alignment of the full-length amino acid sequences of 13 *L. barbarum F-box* genes, a homology of up to 58.46% was observed, with 63.74% identity in the N-terminal and 57.13% in the C-terminal. The sequence alignment revealed that all the genes possess a unique N-terminal (F-box domain) consisting of 42 amino acid residues, and a more distinct F-box-associated domain in the C-terminal.

### 3.2. Similarity in the Structure of F-Box Genes at the S Locus in Angiosperms

The conserved motifs and structures of the 13 S_2_-LbSLFs proteins suggest that they all possess an F-box domain at their N-termini and an F-box-related domain (F_box_assoc_1 superfamily domain) at their C-termini, aligning with observations of F-box domains in other species, such as apples [46], pears [47], and tobacco [30]. Furthermore, all 13 members of the S_2_-LbSLFs family lack introns, corroborating the findings of the study by Wu et al. on *P. inflata. F-box* genes [48]. The gene structure without introns is conducive to the rapid expression of mRNA [49]. Akash et al. found that 31.8% of the *F-box* genes in tomatoes lack introns, indicating that the prevalence of gene structures without introns is a unique characteristic of this F-box family [50].

Cis-regulatory elements are essential in plant regulatory networks and offer insights into the functions of related genes [51]. They have been identified as candidates involved in salt tolerance [52], the response to low-temperature stress [49], and drought induction [53]. In this study, we analyzed the cis-regulatory elements within the 2000-base-pair upstream promoter regions of *S_2_-LbSLFs*. A variety of light-response elements and various elements responsive to hormone reactions were detected. We also identified cis elements related to abiotic stress, such as those involved in defense and stress responses, as well as elements involved in drought induction and binding sites for MYB. Moreover, elements related to plant growth and development, such as those involved in cell cycle regulation, expression in endosperm, and in the expression of meristematic tissues, deserve special attention. In plants, genes containing F-box domains have been shown to regulate plant growth and development [54,55,56] and play important roles in response to adverse stress [57,58]. Therefore, it is speculated that the analysis of cis-regulatory elements may provide new insights into the study of the functions of *S_2_-LbSLF* members, particularly in regulating plant self-incompatibility and related genes, as well as plant development.

### 3.3. S_2_-LbSLF1–S_2_-LbSLF13 Are Good Candidates for the S-Determination Factor of L. barbarum Pollen

The phylogenetic tree analysis reveals that *S_2_-LbSLF1*, *S_2_-LbSLF2, S_2_-LbSLF5*, *S_2_-LbSLF6*, *S_2_-LbSLF7*, and *S_2_-LbSLF3*, *S_2_-LbSLF4*, *S_2_-LbSLF9*, and *S_2_-LbSLF13*, respectively, exhibit a strong clustering relationship with *NtDD6* and *NtDD7*. Wheeler et al. [30] demonstrated that out of the ten SLF proteins associated with tobacco that they identified (referred to as DD1-10), seven are exclusively expressed in pollen. Further meticulous mapping found that *NtDD2*, *NtDD7*, and *NtDD10* are situated in the same chromosomal region as the pollen S-determinant, with *NtDD10* also expressed in petals. Since all the *SLF* genes characterized to date are limited to pollen expression, *NtDD10* can be discounted, suggesting that *NtDD2* and *NtDD7* may function as direct homologs of SLF. In this study, the functions of these nine genes are presumed to be analogous to those of *NtDD6* and *NtDD7*, potentially acting as pollen S-determinants to regulate the self-incompatibility mechanism of *L. barbarum*. Three *L. barbarum* SLF members (*S_2_-LbSLF8*, *S_2_-LbSLF11*, and *S_2_-LbSLF12*) in Group II and four *P. inflata* SLF members cluster together, while Group V encompasses one *L. barbarum SLF* gene (*S_2_-LbSLF10*) and three *P. inflata* SLF members, with *S_2_-LbSLF10* closely associated with *EF614187* and grouped on the same branch. Previously, using RNA-seq, 17 *SLF* genes (*S_2_-SLF1* to *S_2_-SLF17*) and one *SLFLike* gene (*S_2_-SLFLike1*) were identified as being specifically expressed in the pollen transcriptome of *P. inflata* [36]. In this study, the involved genes include (*S_2_-SLF3* to *S_2_*-*SLF6* and *S_2_-SLF11* to *S_2_-SLF13)*, and *S_2_-LbSLF8*, *S_2_-LbSLF10*, *S_2_-LbSLF11*, and *S_2_-LbSLF12* are clustered together with these genes. Overall, these findings indicate that these 13 SLF members can serve as excellent candidates representing the S-determination factors of *L. barbarum* pollen. These *SLF* genes are specifically expressed in *L. barbarum* pollen, aligning with one of the fundamental characteristics of pollen S genes [24,25]. Therefore, *S2-LbSLF1–S2-LbSLF13* are further considered as potential pollen determinants and may contribute to self-incompatibility reactions.

### 3.4. Physical Interactions between S_2_-LbSLFs and S-RNase

Sun et al. have shown through loss-of-function experiments on phlox flowers that the complete set of F-box proteins is the determinant for pollen specificity, confirming that SLF proteins are the sole cause of pollen self-incompatibility (SI) and revealing their interaction with S-RNase to maintain SI [59]. The yeast two-hybrid assay results from this study indicated that the C-terminal regions of *S_2_-LbSLF2*, *S_2_-LbSLF5*, *S_2_-LbSLF6*, *S_2_-LbSLF7*, and the full-length *S_2_-LbSLF2* all exhibited weak interactions with *S_2_-RNase*. Furthermore, the C-terminal region of *S_2_-LbSLF7* demonstrated a weak interaction with *S_5_-RNase*. Therefore, the 13 identified *SLF* genes were tested for point-to-point interactions with *S-RNase* using yeast two-hybrid assays. The results showed that the C-terminal regions of *S_2_-LbSLF2*, *S_2_-LbSLF5*, *S_2_-LbSLF6*, *S_2_-LbSLF7*, and the full-length *S_2_-LbSLF2* could weakly interact with *S_2_-RNase*; the C-terminal region of *S_2_-LbSLF7* could weakly interact with *S_5_-RNase*, indicating that *S_2_-LbSLFs* can interact with *S-RNase* as pollen S-determinant clusters. Qiao et al. have shown that the C-terminal region of *AhSLF*-*S_2_* specifically interacts with S-RNase in Antirrhinum [14]. A similar phenomenon was also observed in phlox, where Liu et al. demonstrated that the C-terminal region of *PhS_3L_*-*SLF1* interacts with *PhS3-RNase*, *PhS3L-RNase*, and *PhSv-RNase* in yeast, while the C-terminal region of *PhSv-SLF1* only interacts with *PhSv-RNase* [19]. Xu et al. also discovered, in their study on the interaction between pear *PbSLFs* and *PbS-RNases*, that *PbS_21_-RNase* interacts with the C-terminal regions of *PbSLF3-S_34_* and *PbSLF6-S_21_*, while *PbS_34_-RNases* only interact with the C-terminal region of *PbSLF3-S_34_* [60]. This is consistent with the results of this study, in which it was found that *S_2_*-*RNase* specifically interacts with the C-terminal regions of *S_2_-LbSLF5*, *S_2_-LbSLF6*, and *S_2_-LbSLF7*, and that *S_5_*-*RNase* only specifically interacts with the C-terminal region of *S_2_-LbSLF7*. This suggests that these interaction relationships depend on the C-terminal regions of *S_2_-LbSLFs* without the F-box domain, rather than the full-length *S_2_-LbSLFs* and their F-box-domain-containing N-terminal regions. This is in line with the function of the C-terminal domain of F-box proteins in the SCF complex, which is responsible for recognizing specific substrate proteins. However, a difference in this study is the observation that the full-length *S_2_-LbSLF2* can also interact with *S_2_-RNase*.

Regarding the self-incompatibility (SI) mechanism of *P. inflata*, numerous studies have indicated that the S-locus factor (*SLF*) interacts with non-self *S-RNase* and specifically neutralizes it [16,35,41,61]. Nonetheless, Liu et al. conducted a yeast double-hybrid assay to study the interaction between *SLF* and *S-RNase* in *P. hybrida*, revealing that *PhSLF* can specifically engage with both “self” and “non-self” *PhS-RNase* [19]. Moreover, in *P. inflata*, the interaction between *PiSLF* and non-self *PiS-RNase* is more potent than that with the self *S-RNase* [62]. The current study demonstrates that only *S_2_-LbSLF7* can interact with non-self *S_5_-RNase*, while most of the interaction outcomes involve *S_2_-LbSLF* binding to the self *S_2_-RNase*, a pattern that contrasts with the majority of the interactions observed in other Solanaceae plants. Additionally, research on Rosaceae and Plantaginaceae has also uncovered interactions between *SLF* and both “self” and “non-self” *S-RNase*. For example, Yuan et al. reported in their study on apples that the *S2-MdSFBB1* in apples is capable of interacting with both *S_2_-* and *S_3_-RNases*, *S2-MdSFBB2* interacted with *S_7_-RNase*, and both *S2-MdSFBB3* and *S2-MdSFBB4* had the capacity to interact with *S_3_-*, *S_5_-* and *S_9_-RNases*. In addition [63], Qiao et al. utilized yeast two-hybrid tests and co-immunoprecipitation to show that *AhSLF-S_2_* interacts with *S_2_-*, *S_4_-*, and *S_5_-RNases* in *Antirrhinum* [64].

In conclusion, the data suggest that *S_2_-LbSLFs* can selectively interact with both “self” and “non-self” *S-RNases* in yeast cells. In other Solanaceous plants, *S-RNase* functions according to a non-self-recognition pattern, meaning that *SLFs* interact with *S-RNases* that are not of their own type. In the case of *L. barbarum*, *S_2_-LbSLFs* engage in interactions not only with *S_2_-RNase*, but also with *S_5_-RNase*. Additionally, *S_2_-LbSLF* shows a more robust interaction with its cognate *S-RNase* than with non-self *S-RNase*. Consequently, the relationship between *S-RNase* and *SLF* may involve intricate interplay.

## 4. Materials and Methods

### 4.1. Identification of the L. barbarum F-Box Gene Family

The genomic data for *L. barbarum* were retrieved from the NCBI online database (https://www.ncbi.nlm.nih.gov/ (accessed on 20 March 2023)). An HMM (PF00646) file corresponding to the *F-box* gene domain was obtained from the Pfam protein database (http://www.pfam.xfam.org/ (accessed on 20 March 2023)). This F-box HMM file was then used with HMMER 3.0 software to identify the entire *F-box* gene family within the *L. barbarum* protein sequences, with the E-value threshold set at 1 × 10^−5^. The proteins identified were subsequently submitted to the NCBI-CDD (https://www.ncbi.nlm.nih.gov/cdd/ (accessed on 20 March 2023)), SMART (http://smart.embl.de/ (accessed on 20 March 2023)), and Pfam (http://pfam.xfam.org/ (accessed on 20 March 2023)) online databases for conservation domain analysis to verify the presence of the F-box domain. Next, differential expression analysis was performed on all *F-box* genes located on chromosome 2, leading to the identification of the gene members that were specifically expressed in the stamen on that chromosome.

### 4.2. Gene-Specific Amplification and Sequence Analysis

The full-length clone of the candidate gene was performed. Genomic DNA was extracted from the leaves of Ningqi 1 (S_2_S_8_) using a novel plant genome DNA-extraction kit (Tiangen, Beijing, China), and was stored for future applications. Following the haploid genome data of Ningqi 1 (PRJNA640228), specific forward and reverse primers for the *SLF* gene were designed based on the sequence flanking the *S_2_-RNase* gene located in this study. Primers for the *SLF* gene were designed based on the predicted sequences derived from genomic sequencing (refer to Supplementary Table S2 for primer details). The PCR amplification was conducted using a 2× Hieff Canace^®^ Gold PCR Master Mix high-fidelity enzyme pre-mix (Yi Sheng, Shanghai, China) with a PCR system containing 100 ng of DNA template, 0.5 μM forward and reverse primers, and 1× Hieff Canace^®®^ Gold PCR Master Mix. The PCR cycling conditions included 5 min of pre-denaturation at 94 °C, 30 s denaturation at 94 °C, 30 s annealing at 60 °C, 45 s extension at 72 °C, and 35 cycles, followed by a final 10-min extension at 72 °C. The PCR products were then separated using 1% agarose gel electrophoresis, and the purified fragments were ligated into the Hieff Clone ZeroTOPO-Blunt Cloning Kit (Yi Sheng, Shanghai, China) vector before being transformed into DH5α competent cells. These transformed cells were sent to the Beijing Uniquehexa Genetic Technology Limited Research Center for sequencing. Sequence-alignment analysis was conducted using DNAMAN software (v10) (the F-box protein sequences of *L. barbarum* are listed in Supplementary Table S3).

### 4.3. Physicochemical Property Analysis of S_2_-LbSLFs Genes

The protein sequences of 13 *SLF* genes were analyzed using the ExPASy online platform (https://web.expasy.org/pr-otparam/ (accessed on 12 April 2023)) to determine the number of amino acids, molecular weight, isoelectric point, and hydrophobicity of the proteins. The secondary structure of SLF proteins was analyzed using the SOPMA online platform (https://npsa-prabi.ibcp.fr/cgi-bin/npsa_automat.pl?pag-e=npsa_sopma.html/ (accessed on 12 April 2023)). The subcellular localization of S_2_-LbSLFs proteins was predicted using Cell-PLoc 2.0 software.

### 4.4. Phylogenetic Tree Development Analysis

To explore the evolutionary relationships of *F-box* genes within and between species, protein sequences from 10 tobacco *F-box* genes, 15 morning glory *F-box* genes, and 13 identified *L. barbarum* F-box proteins were retrieved from NCBI (https://www.ncbi.nlm.nih.gov/ (accessed on 15 April 2023)) and underwent multiple sequence alignments (the F-box protein sequences of each species are listed in Supplementary Table S3). Clustering was executed employing a distance matrix strategy, utilizing the Poisson correction model for calculations (d = −ln(1 − p), where d signifies the genetic distance between protein sequences and p represents the percentage of sequence variation) [65]. Subsequently, the neighbor-joining method (NJ) [66] from MEGA11 was applied to amalgamate sequences into a singular node based on the shortest distances within the distance matrix, leading to the assembly of a comprehensive phylogenetic tree. This approach was supplemented by the bootstrap method, which generated replicated samples across various datasets, with a bootstrap replication count of 1000, to evaluate the integrity of the constructed phylogenetic tree [67]. The ITOL (https://itol.embl.de/ (accessed on 15 April 2023)) online tool was then used to visualize the evolutionary tree.

### 4.5. Analysis of Conserved Protein Motifs, Protein Domains, and Gene Structure

The exon distributions of the *S_2_-LbSLFs* genes were extracted from the GFF file corresponding to the *L. barbarum* genome. Default settings were employed in the Tbtools-Simple MEME Wrapper, Tbtools-Visualize Gene Structure, and NCBI-CCD databases to analyze the conserved motifs, gene structures (exons and introns), and protein domains for the *S_2_-LbSLFs*. Subsequent visualization was carried out using the Tbtools-Simple Biosequence Viewer.

### 4.6. Analysis of Cis Elements in SLF Gene Family Members

To explore the potential regulation of *S_2_-LbSLFs*, a 2000-base-pair sequence upstream of the ATG start codon for *S_2_-LbSLF* genes was isolated from the *L. barbarum* genome and considered as the promoter region. This sequence was then uploaded to the PlantCARE webtool (http://bioinformatics.psb.ugent.be/webtools/PlantCARE/html/ (accessed on 6 May 2023)) for the prediction of cis-regulatory elements within the promoter. Statistical analyses of the predictions were conducted using Microsoft Excel 2019, and heatmaps were created with TBtools (v2.042) software. These heatmaps were later refined using Adobe Illustrator CC 2019.

### 4.7. Analysis of S_2_-LbSLFs Gene Density and Collinearity

Utilizing the *L. barbarum* genome files and GFF annotations, the One Step MC ScanX feature in Tbtools was utilized to perform gene duplication and collinearity analysis on *L. barbarum*. Advanced Circos was employed to create circular diagrams that depicted the collinear relationships. The collinearity analysis of the *S_2_-LbSLFs* was carried out using Tbtools’ One Step MC ScanX-Supper Fast tool.

### 4.8. RNA Extraction and Expression Patterns of S_2_-LbSLFs

To investigate the expression patterns of the *F-box* gene family, we isolated *F-box* genes situated on chromosome 2 and created heatmaps of their expression profiles using TBtools (v2.042) software, which is based on transcriptomic data. To validate the accuracy of the expression levels inferred from the transcriptomic data, we then conducted qRT-PCR analysis. Specimens of *L. barbarum* ‘Ningqi 1’ were harvested from the Goji Germplasm Resource Nursery of Yinchuan Botanical Garden (38°24′ N, 106°10′ E), encompassing leaves, petals, entire flowers, stigma, anthers, and flower stems. These samples were rapidly frozen in liquid nitrogen and subsequently stored in a freezer at −80 °C for RNA extraction and subsequent fluorescence quantitation analysis. The RNA extraction from the plant samples was performed using the method described by Chen et al. [68], and the concentration and purity of the RNA were assessed using a Nano-500B microspectrophotometer (Aosheng, Shanghai, China). Subsequently, the RNA was reverse-transcribed into cDNA using the PrimeScript RT reagent kit with gDNA Eraser (Takara, Dalian, China). Thirteen qRT-PCR primers designed for *S_2_-LbSLFs* were created using Primer 5.0, with Actin serving as the internal control gene (the primers for qRT-PCR are detailed in Supplementary Table S4). Real-time fluorescence quantitation was carried out using TB Green^®®^ Premix Ex Taq™ II (Takara, Dalian, China) with a reaction mixture containing 100 ng of cDNA template, 1× TB Green Premix Ex Taq II, and 0.4 μM of forward and reverse primers, supplemented with ddH_2_O for a final volume of 20 μL. The reaction protocol involved a 30-s pre-denaturation at 95 °C a 5-s denaturation at 95 °C, a 30-s annealing at 60 °C, and 40 cycles of denaturation and annealing. Each experiment was replicated with three biological samples, and the relative expression levels of *S_2_-LbSLFs* genes were analyzed using the 2^−ΔΔCt^ method.

### 4.9. Yeast Two-Hybrid Analysis of Interaction between SLF and S-RNase

To further investigate the interaction between *S_2_-LbSLFs* and *S-RNases*, a yeast two-hybrid assay was employed. We used *S_2_-LbSLFs* as prey and *S_5_-RNase* and *S_2_-RNase* as bait. The *S_2_-LbSLFs* were divided into full-length SLFs, N-terminal SLFs, and C-terminal SLFs, which were then cloned and ligated into pGADT7(AD). The *S_2_-RNase* and *S_5_-RNase* in their full-length forms were ligated into pGBKT7(BD). Vectors containing different SLF fragments were co-transformed into the yeast Y2Hgold strain with S-RNase for interaction experiments. The transformed yeast cells were grown on -Leu/-Trp agar plates at 30 °C for 4 to 5 days. The colonies were further grown on -Leu/-Trp/-Ade/-His agar plates at 30 °C for an additional 3 to 4 days to test for interactions.

### 4.10. Statistical Analysis

All experiments in this study were conducted with three biological replicates. Data management was facilitated through Excel 2019. To normalize and visualize the transcriptomic data, the logarithm of FPKM values was computed, followed by the generation of clustered heatmaps with the TBtools (v2.042) software. A one-way ANOVA using IBM SPSS Statistics 27.0 was conducted to analyze significant differences, with *p*-values of less than 0.05 deemed statistically significant. The results presented are represented as the mean ± standard error based on the average of the three biological replicates. Graphical representations were produced using GraphPad Prism 9.5.

## 5. Conclusions

In this investigation, we identified and cloned 13 *S_2_-LbSLF* genes from the *L. barbarum* genome. The physicochemical properties of these 13 S_2_-LbSLFs were analyzed, revealing that all the proteins are predicted to be nucleus-localized. The collinearity analysis failed to detect any tandem duplications within the *S_2_-LbSLF* gene family. Two pairs of collinear genes were found in *L. barbarum* and *S. lycopersicum*, respectively, with a single pair in *R. chinensis*. The *S_2_-LbSLFs* were divided into six groups in the phylogenetic analysis, with four genes clustering with *F-box* genes encoding pollen determinants in *P. inflata*, and the remaining nine genes associating with genes specifically expressed in the pollen of *N. tabacum*, suggesting they may serve as promising candidate genes for pollen determinants. These *SLF* genes displayed pollen-specific expression. The yeast two-hybrid assays demonstrated an interaction between S-RNase and the C-terminal region of the SLF. These results pave the way for further studies to elucidate the functional roles of *SLF* genes as pollen determinants regulating self-incompatibility in *L. barbarum*.

## Figures and Tables

**Table 1:** Predicted physiochemical properties of S_2_-LbSLF proteins.

Gene ID	Protein Length (aa)	Molecular Weight (Da)	Theoretical pI	Instability Index	Aliphatic Index	Grand Average of Hydropathicity
*S_2_-LbSLF1*	389	44,852.75	4.81	49.36	93.88	−0.063
*S_2_-LbSLF2*	389	44,951.82	5.28	40.67	89.85	−0.076
*S_2_-LbSLF3*	393	45,512.54	5.74	41.11	94.22	−0.059
*S_2_-LbSLF4*	393	45,805.57	5.12	46.43	89.26	−0.183
*S_2_-LbSLF5*	389	44,812.70	4.84	46.66	92.11	−0.060
*S_2_-LbSLF6*	390	45,250.00	5.38	39.44	91.38	−0.105
*S_2_-LbSLF7*	390	45,187.04	5.92	34.02	90.38	−0.111
*S_2_-LbSLF8*	390	44,949.81	5.07	42.69	99.41	−0.007
*S_2_-LbSLF9*	358	42,018.48	7.07	43.74	87.35	−0.248
*S_2_-LbSLF10*	388	45,029.85	7.06	42.38	86.34	−0.217
*S_2_-LbSLF11*	391	45,278.55	6.32	47.75	91.69	−0.044
*S_2_-LbSLF12*	388	45,351.63	5.23	46.10	93.87	−0.059
*S_2_-LbSLF13*	388	45,351.63	5.23	46.10	93.87	−0.059

**Table 2:** Secondary structure prediction for S_2_-LbSLF proteins.

Gene Name	Subcellular Location	Alpha Helix/%	Extended Strand/%	Beta Turn/%	Random Coil/%
*S_2_-LbSLF1*	Nucleus	17.48	32.13	3.08	47.30
*S_2_-LbSLF2*	Nucleus	18.25	30.59	4.11	47.04
*S_2_-LbSLF3*	Nucleus	15.01	31.30	5.09	48.60
*S_2_-LbSLF4*	Nucleus	19.08	30.03	3.82	47.07
*S_2_-LbSLF5*	Nucleus	16.97	32.65	4.11	46.27
*S_2_-LbSLF6*	Nucleus, Cell membrane	17.18	30.51	3.59	48.72
*S_2_-LbSLF7*	Nucleus	19.23	29.74	4.10	46.92
*S_2_-LbSLF8*	Nucleus	17.44	30.00	4.62	47.95
*S_2_-LbSLF9*	Nucleus	16.20	30.73	4.47	48.60
*S_2_-LbSLF10*	Nucleus	18.56	28.09	4.90	48.45
*S_2_-LbSLF11*	Nucleus, Cell membrane, Chloroplast	18.16	30.18	4.09	47.57
*S_2_-LbSLF12*	Nucleus	17.01	30.67	4.64	47.68
*S_2_-LbSLF13*	Nucleus	15.27	30.28	4.07	50.38

## Data Availability

The datasets supporting the results presented in this manuscript are included within the article (and its Supplementary Materials).

## Supplementary Materials

The following supporting information can be downloaded at: https://www.mdpi.com/article/10.3390/plants13070959/s1: Supplementary Table S1. Binding site prediction results of *S_2_-LbSLFs* promoters; Supplementary Table S2. Cloning primers; Supplementary Table S3. Protein sequence of SLFs from *L. barbarum*, *N. tabacum*, and *P. inflata*; Supplementary Table S4. The qRT-PCR primers; Supplementary Figure S1. The interaction between S_2_-RNase and S_2_-LbSLFf was investigated in yeast strains; Supplementary Figure S2. The interaction between S_2_-RNase and S_2_-LbSLFn was investigated in yeast strains; Supplementary Figure S3. The interaction between S_2_-RNase and S_2_-LbSLFc was investigated in yeast strains; Supplementary Figure S4. The interaction between S_5_-RNase and S_2_-LbSLFf was investigated in yeast strains; Supplementary Figure S5. The interaction between S_5_-RNase and S_2_-LbSLFn was investigated in yeast strains; Supplementary Figure S6. The interaction between S_5_-RNase and S_2_-LbSLFc was investigated in yeast strains.
