# Supplementary material for: The Identification and Analysis of the Self-Incompatibility Pollen Determinant Factor SLF in Lycium barbarum
Source: Plants (Basel). 2024 Mar 26;13(7):959. doi: 10.3390/plants13070959 (PMC11013074; doi:10.3390/plants13070959)
Supplement: Supplementary file 1 [file plants-13-00959-s001.zip › Supplementary Figure S3. The interaction between S2-RNase with S2-LbSLFc was investigated in yeast strains.pdf]

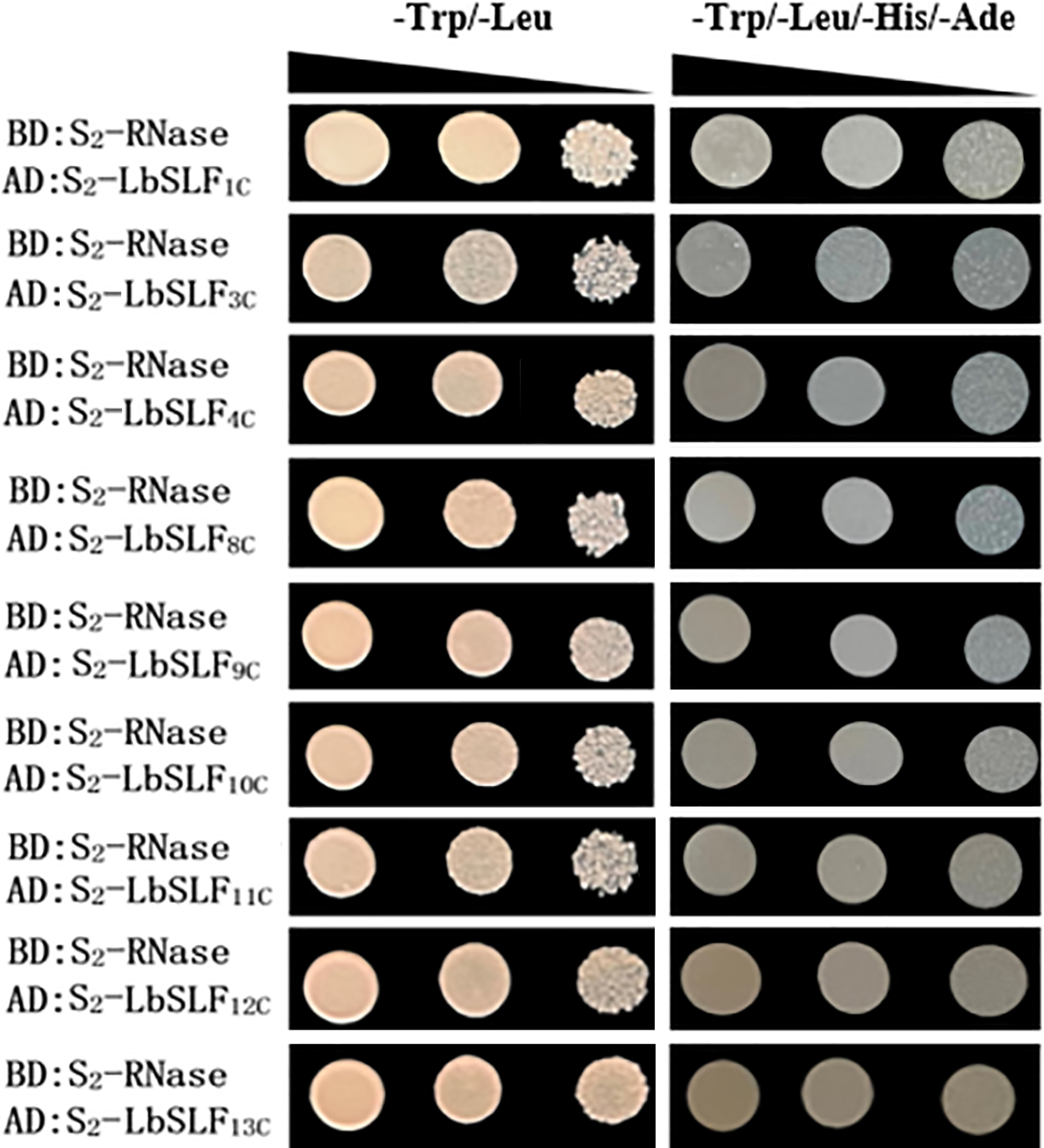
-Trp/-Leu
-Trp/-Leu/-His/-Ade
BD:S2-RNase
AD:S2-LbSLF1C
BD:S2-RNase
AD:S2-LbSLF3C
BD:S2-RNase
AD:S2-LbSLF4C
BD:S2-RNase
AD:S2-LbSLF8C
BD:S2-RNase
AD:S2-LbSLF9C
BD:S2-RNase
AD:S2-LbSLF10C
BD:S2-RNase
AD:S2-LbSLF11C
BD:S2-RNase
AD:S2-LbSLF12C
BD:S2-RNase
AD:S2-LbSLF13C
