# Supplementary material for: The Identification and Analysis of the Self-Incompatibility Pollen Determinant Factor SLF in Lycium barbarum
Source: Plants (Basel). 2024 Mar 26;13(7):959. doi: 10.3390/plants13070959 (PMC11013074; doi:10.3390/plants13070959)
Supplement: Supplementary file 1 [file plants-13-00959-s001.zip › Supplementary Figure S4. The interaction between S5-RNase with S2-LbSLFf was investigated in yeast strains.pdf]

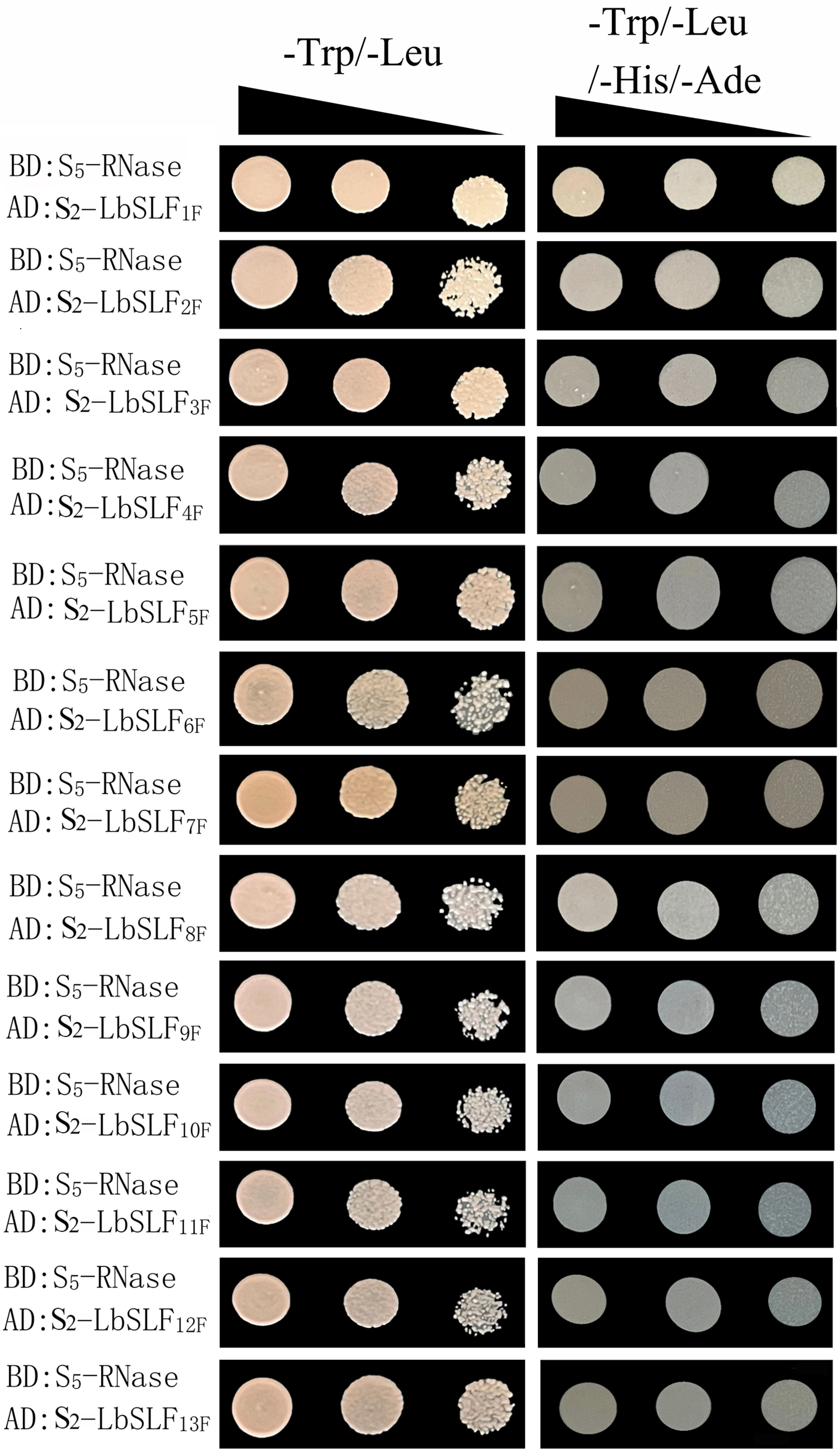
-Trp/-Leu
-Trp/-Leu
/-His/-Ade
BD：S_5-RNase
AD：S_2-LbSLF$_{1F}$
BD：S_5-RNase
AD：S_2-LbSLF$_{2F}$
BD：S_5-RNase
AD： S_2-LbSLF$_{3F}$
BD：S_5-RNase
AD：S_2-LbSLF$_{4F}$
BD：S_5-RNase
AD： S_2-LbSLF$_{5F}$
BD：S_5-RNase
AD：S_2-LbSLF$_{6F}$
BD：S_5-RNase
AD： S_2-LbSLF$_{7F}$
BD：S_5-RNase
AD： S_2-LbSLF$_{8F}$
BD：S_5-RNase
AD： S_2-LbSLF$_{9F}$
BD：S_5-RNase
AD：S_2-LbSLF$_{10F}$
BD：S_5-RNase
AD：S_2-LbSLF$_{11F}$
BD：S_5-RNase
AD：S_2-LbSLF$_{12F}$
BD：S_5-RNase
AD：S_2-LbSLF$_{13F}$
