# Supplementary figures and images for: The Identification and Analysis of the Self-Incompatibility Pollen Determinant Factor SLF in Lycium barbarum
Source: Plants (Basel). 2024 Mar 26;13(7):959. doi: 10.3390/plants13070959 (PMC11013074; doi:10.3390/plants13070959)

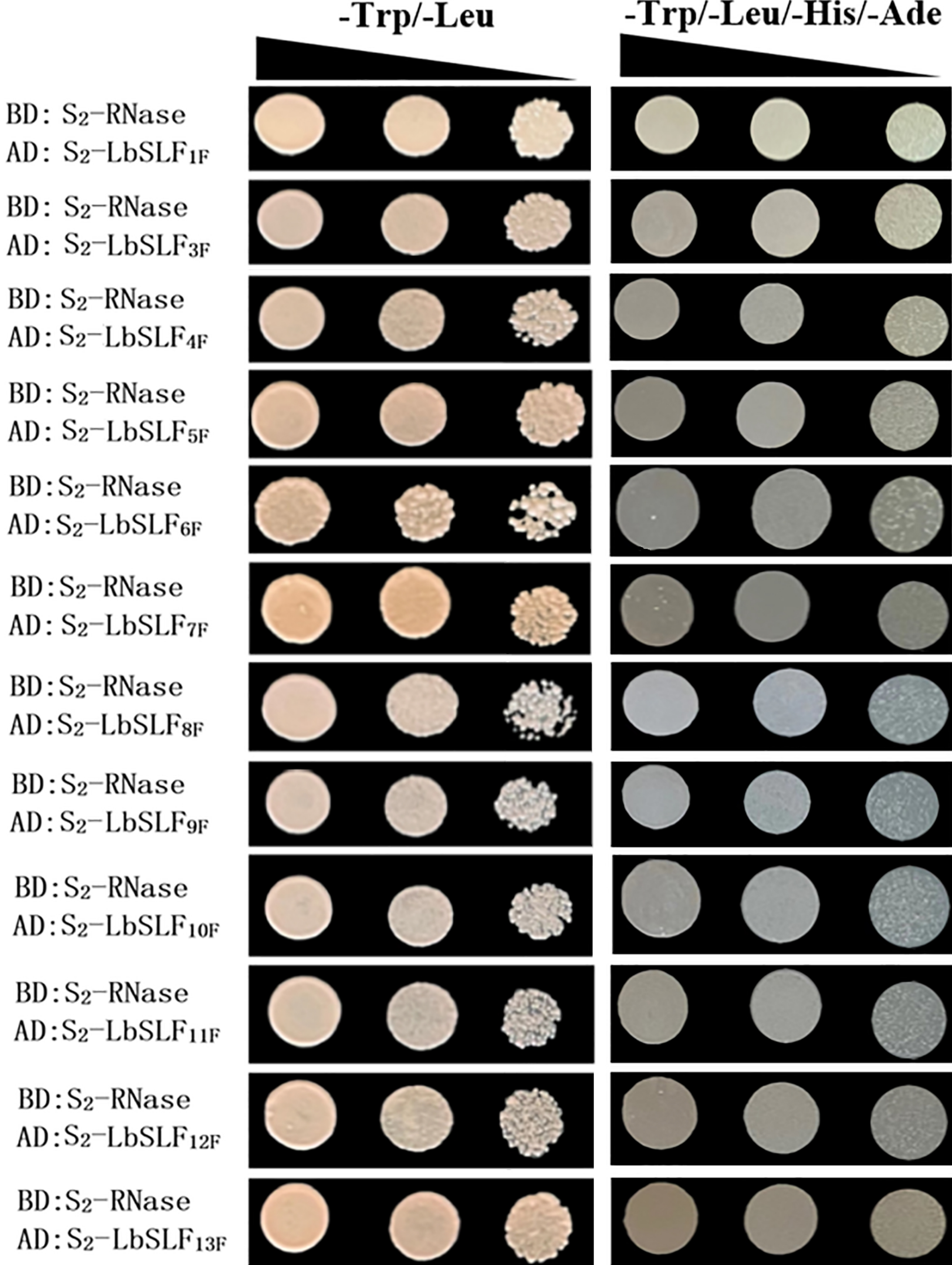

Supplement: Supplementary file 1 [file plants-13-00959-s001.zip › Supplementary Figure S1. The interaction between S2-RNase with S2-LbSLFf was investigated in yeast strains.pdf]

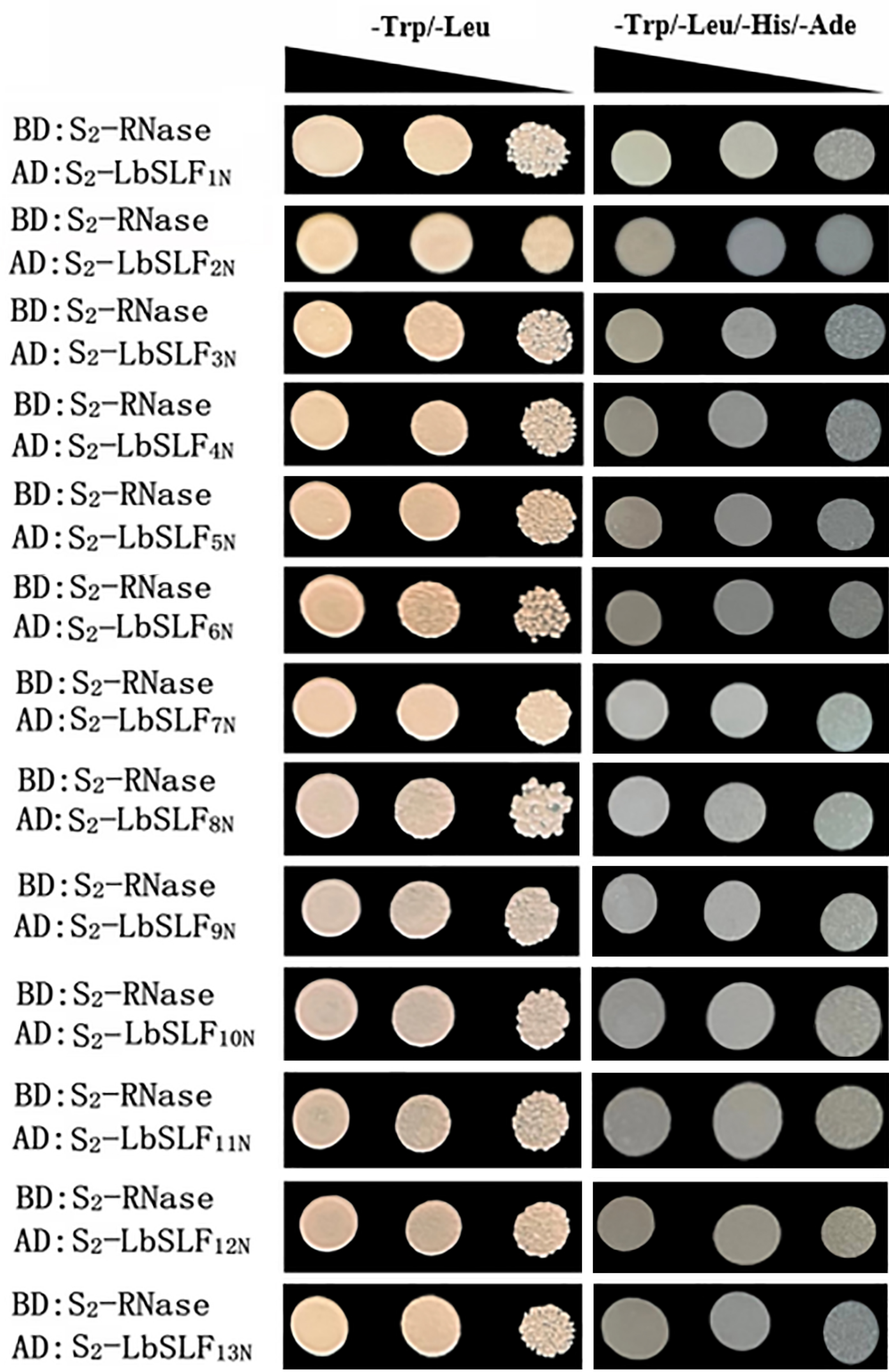

Supplement: Supplementary file 1 [file plants-13-00959-s001.zip › Supplementary Figure S2. The interaction between S2-RNase with S2-LbSLFn was investigated in yeast strains.pdf]

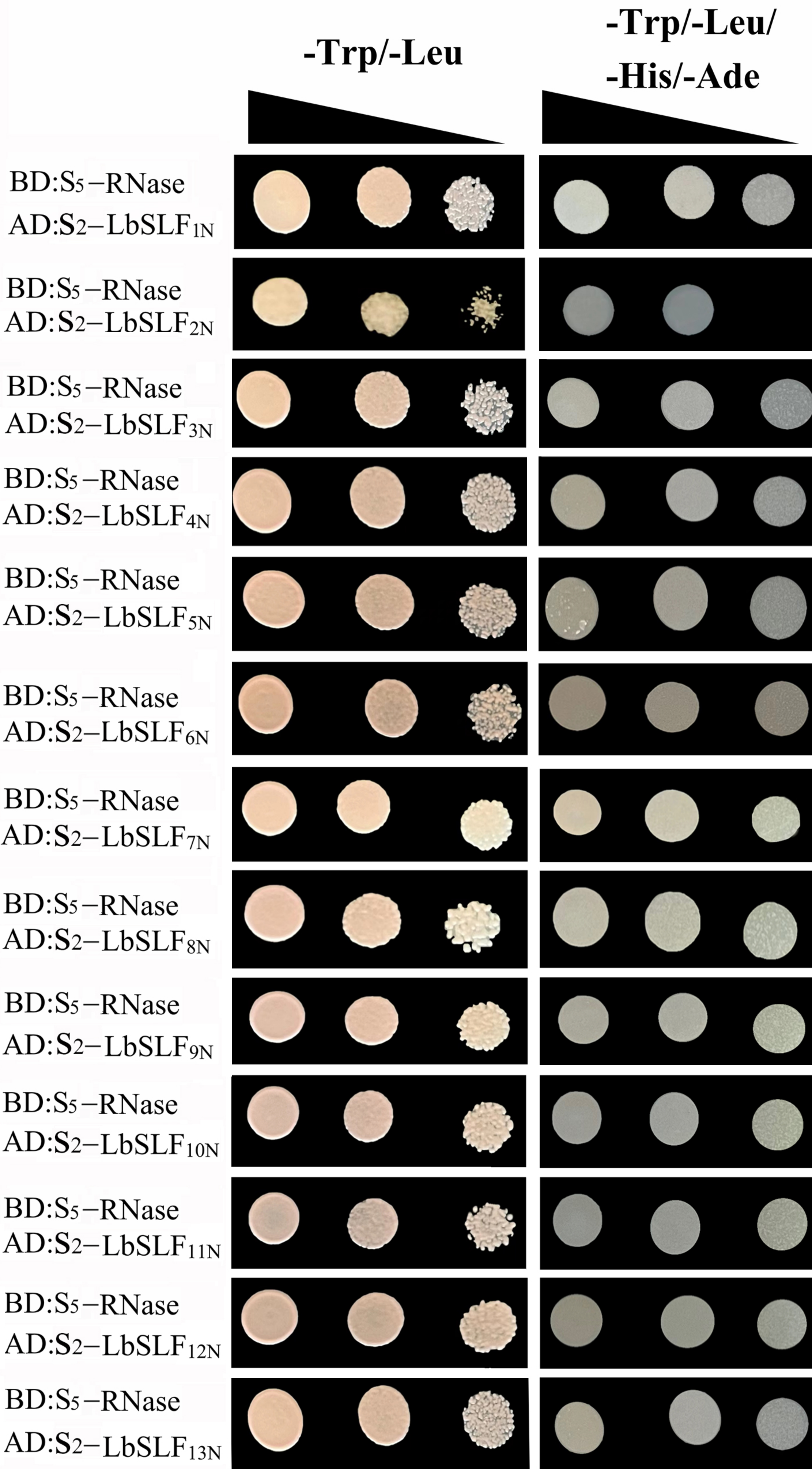

Supplement: Supplementary file 1 [file plants-13-00959-s001.zip › Supplementary Figure S5. The interaction between S5-RNase with S2-LbSLFn was investigated in yeast strains.pdf]

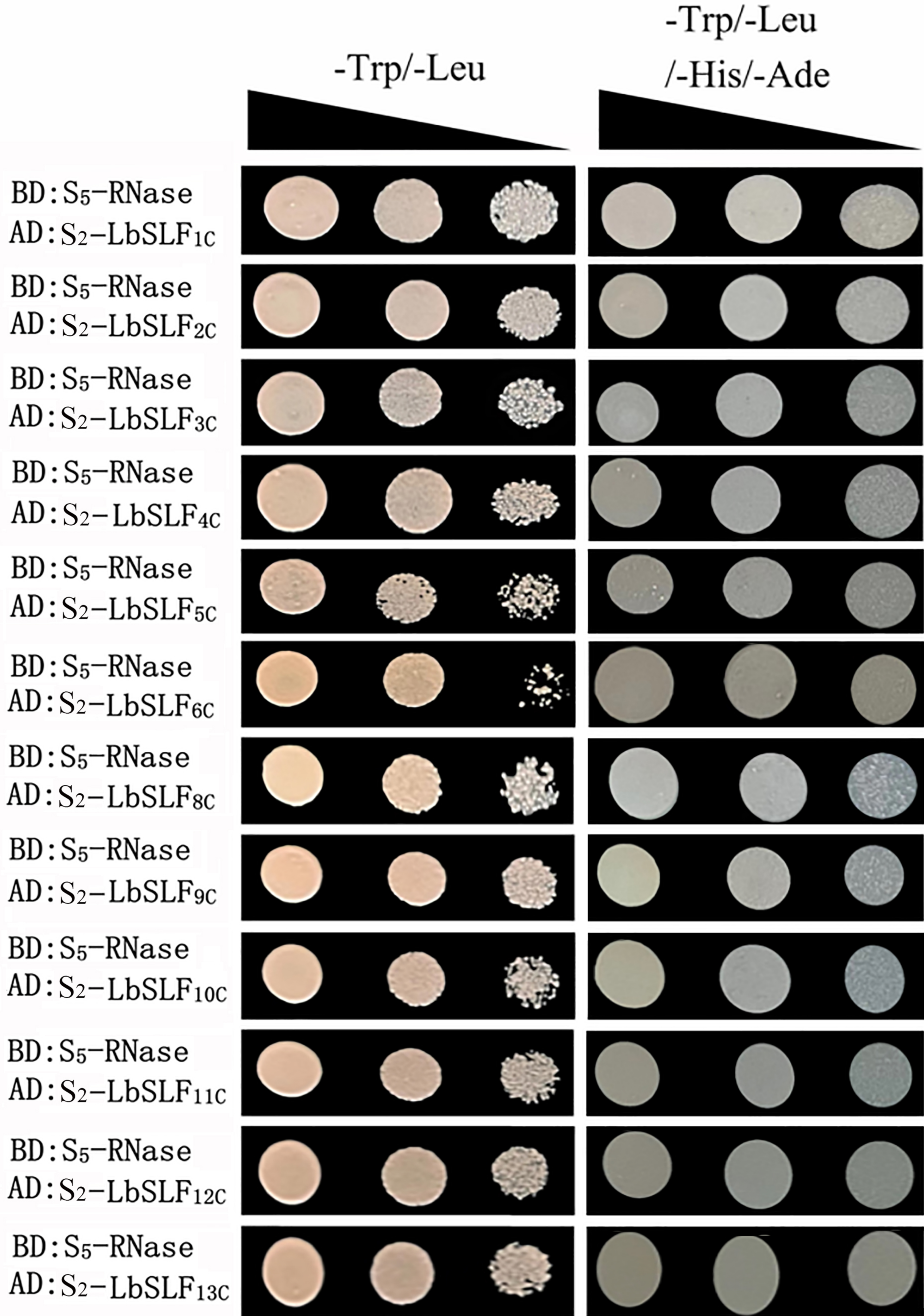

Supplement: Supplementary file 1 [file plants-13-00959-s001.zip › Supplementary Figure S6. The interaction between S5-RNase with S2-LbSLFc was investigated in yeast strains.pdf]
